# High-Efficiency Surface-Cooled Rapid Tooling Development for Injection Molding of Low-Density Polyethylene

**DOI:** 10.3390/polym17040468

**Published:** 2025-02-11

**Authors:** Chil-Chyuan Kuo, Pin-Han Lin, Jing-Yan Xu, Armaan Farooqui, Song-Hua Huang

**Affiliations:** 1 Department of Mechanical Engineering, Ming Chi University of Technology, No. 84, Gungjuan Road, New Taipei City 24301, Taiwan; 2 Department of Mechanical Engineering, Chang Gung University, No.259, Wenhua 1st Rd., Guishan Dist., Taoyuan City 33302, Taiwan; 3 Department of Mechanical Engineering, Chhattisgarh Swami Vivekanand Technical University, Bhilai 491107, Chhattisgarh, India; 4 Li-Yin Technology Co., Ltd., No. 37, Lane 151, Section 1, Zhongxing Road, Wugu District, New Taipei City 24872, Taiwan

**Keywords:** epoxy resin filled with aluminum particles, polymer composite material, surface-cooled cooling channels, plastic injection molding, heat dissipation time, Moldex3D simulation

## Abstract

Epoxy resin filled with aluminum particles constitutes a polymer composite material commonly utilized in research and development departments to fabricate rapid tooling for prototyping new designs. This study developed aluminum-filled epoxy resin molds by incorporating surface-cooled cooling channels (SCCCs) to enhance cooling performance, validated through Moldex3D simulation and experimental analysis. The simulation revealed that a 1 mm mesh size was utilized to balance accuracy and efficiency, with simulations revealing the complete filling of the injection-molded product within 5 s. This study examines rapid tooling with surface-cooled cooling channels in low-density polyethylene injection molding. The reliable parameters include a melt temperature of 160 °C, a mold temperature of 30 °C, an injection pressure of 10 MPa, and a heat dissipation time of 20 s. These parameters effectively minimize the risk of mold cracking while ensuring efficient molding. The SCCC demonstrates superior cooling performance, enhancing cooling efficiency by 58.7% compared to the conventional conformal cooling channel. It reduces cooling time, enhances production capacity, and shortens delivery times. Additionally, it lowers energy consumption, carbon emissions, and the rate of product defects in large-scale manufacturing. A cooling mechanism of SCCC after LDPE injection molding was also proposed.

## 1. Introduction

Plastic injection molding [1,2,3] has several significant benefits. First, it enables fast production after mold setup, making it well-suited for high-volume manufacturing. The process allows for the production of complex, intricate parts that would be challenging with other techniques. A broad selection of plastics, including thermoplastics and elastomers, can be utilized. Moreover, material waste is kept to a minimum, and excess plastic can often be reused. The method ensures consistent part quality with minimal variation. For large-scale manufacturing, it is highly cost-efficient, reducing individual part costs and offering excellent economic value for mass production projects. Aluminum-filled epoxy resin [4] belongs to the class of polymer materials. Epoxy resins are thermosetting polymers that are utilized in multiple fields due to their superior mechanical strength, excellent adhesion properties, and high chemical resistance. Incorporating aluminum particles into epoxy resin creates an aluminum-filled epoxy composite, which retains the inherent properties of the epoxy resin while benefiting from enhanced mechanical strength, thermal conductivity, and electrical performance due to the aluminum fillers.

Lai et al. [5] conducted a mold flow analysis and found that using conformal and baffle cooling channels improved the internal temperature by 8.1%, reduced the mold temperature by 11.2%, and decreased the volume shrinkage rate by 8.2%, enhancing product quality and cycle efficiency. Wang et al. [6] introduced an origami-inspired method for three-dimensional topology optimization by applying two-dimensional techniques to design conformal cooling channels, achieving a 6.7 K reduction in casting temperature and an 11.06% decrease in pressure drop for hexahedral castings. Kariminejad et al. [7] replaced straight-drilled cooling channels with conformal cooling channels in a sensorized mold for complex industrial components, achieving a 50% reduction in production cycle time and improved component quality. Li et al. [8] demonstrated that an optimized series flat-head conformal cooling channel reduced the injection molding cycle by 62.43%, highlighting the significant impact of cooling system design on production efficiency for deep cavity shells. Gao et al. [9] suggested that the optimal Reynolds number for conformal cooling channels is 20,000, contrasting with the previously proposed value of 100,000, to avoid excessive pressure drops while ensuring adequate cooling performance. Wilson et al. [10] demonstrated that a generative design-optimized system significantly outperforms both manually designed and non-conformal tools, achieving up to 70% faster cooling and reducing cycle time and part warpage. Chen et al. [11] proposed a novel calculation formula that accounts for its thermal properties, resulting in high cooling efficiency at a 0.10 mm thickness and revealing a 2% decrease in cooling efficiency with variable thicknesses. Chung and Liu [12] innovatively combined metal injection molding (MIM) with metal additive manufacturing (MAM) to assess the impact of conformal cooling channels, revealing a 92.8% reduction in mold preheating time, a 15.46% improvement in cavity temperature uniformity. Mao and Chen [13] found that multi-micro parallel conformal channels enhanced cooling efficiency, reduced the molding cycle by 23.3%, and minimized the temperature difference on the mold surface to within 1.2 °C. Guanhua et al. [14] demonstrated that these TPMS-based conformal cooling channels for an automotive hood cover outperform traditional conformal channels, achieving an 8.48 K reduction in average cooling surface temperature and a 24.65% decrease in temperature distribution variability. CCC [15] has gained widespread application in the injection molding industry due to its ability to provide consistent cooling following the filling stage [16], especially during the cooling phase [17,18]. Despite its advantages, conventional CCCs fabricated via direct metal laser sintering have been found to experience sagging issues [19]. Furthermore, a key shortcoming of CCC is the pronounced pressure drop across the cooling channels.

Low-density polyethylene (LDPE) resins [20] are a type of versatile thermoplastic valued for their flexibility, chemical resistance, and low cost. It is commonly used in packaging, electrical insulation, industrial liners, and construction as a moisture barrier [21]. According to practical experience, the CCC [22] sees widespread application in injection molding due to its ability to deliver steady cooling post-filling [23] during the heat dissipation stage [24,25]. Unfortunately, the conventional CCC, manufactured using direct metal laser sintering, exhibits a sagging effect [26]. Furthermore, CCC faces a notable drawback: significant pressure loss along the cooling channels. To overcome this challenge, the current study proposes a novel solution named surface-cooled cooling channel (SCCC).

Aluminum-filled epoxy resin is a polymer composite material combining epoxy resin as the matrix and aluminum powder as the filler to enhance thermal, electrical, and mechanical properties for diverse industrial applications. An aluminum-filled epoxy resin rapid tooling method incorporating an SCCC system was developed and implemented in practical applications. The cooling efficiency of the fabricated tooling was assessed via LDPE plastic injection molding. To address this challenge, this study proposes a novel SCCC design, with an injection mold constructed using aluminum-filled epoxy resin and integrated with the SCCC system, which was subsequently designed and experimentally validated. Two kinds of aluminum-filled epoxy resin molds were fabricated, and the heat dissipation time for the molded component was evaluated using a plastic injection molding machine. A fast prototype featuring SCCC was developed to mitigate pressure drop issues using the Moldex3D simulation software [27]. Finally, this study established a database of high cooling efficiency rapid tooling for injection molding of LDPE.

## 2. Materials and Methods

**Figure 1:**
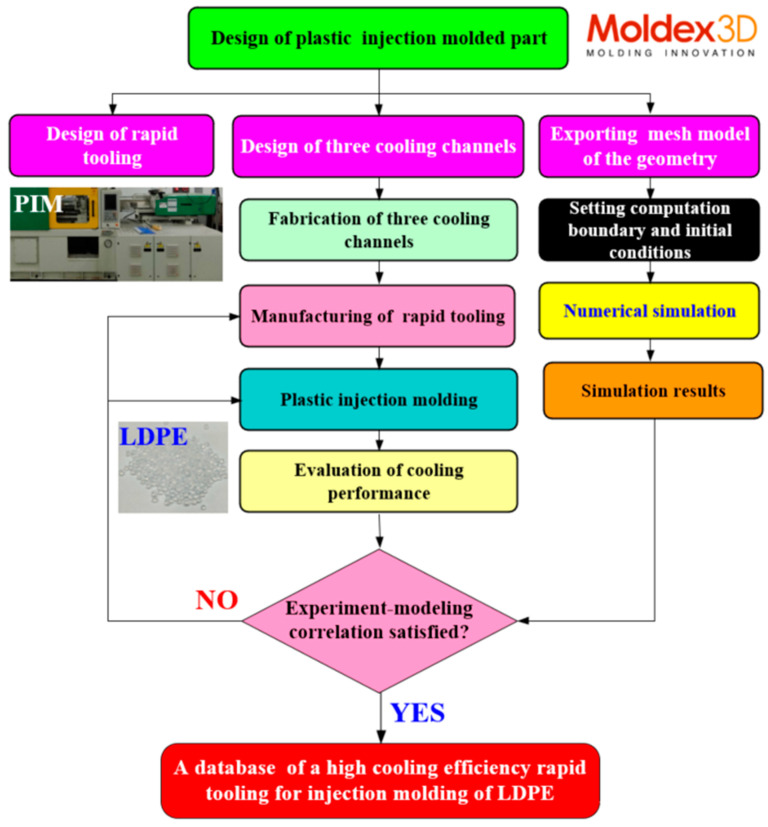
Steps in the research methodology.

**Figure 2:**
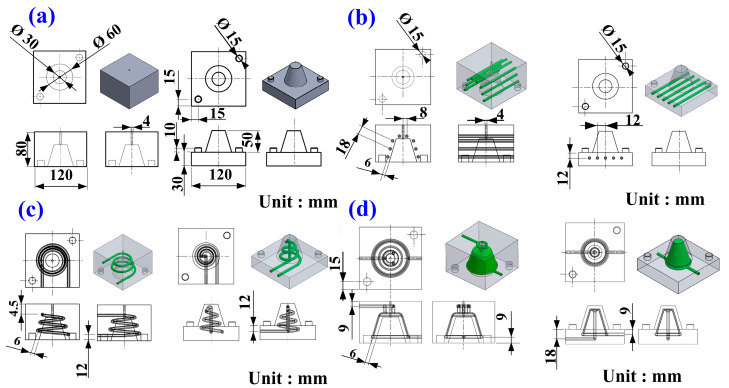
Three-dimensional visualization and dimensions of the fast prototype (**a**) without a cooling channel, (**b**) with the conventional cooling channel, (**c**) with the conformal cooling channel, and (**d**) with the surface-cooled cooling channel.

**Figure 3:**
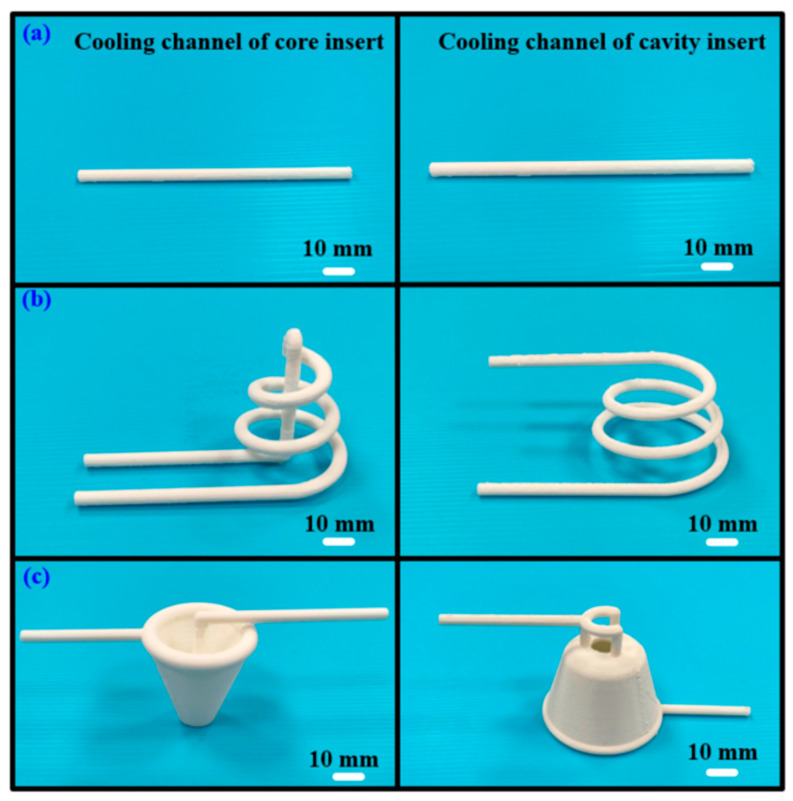
Cooling channels after removing the support material. (**a**) with conventional cooling channel, (**b**) with conformal cooling channel, and (**c**) with surface-cooled cooling channel.

Figure 1 depicts the schematic representation of the study workflow. The process begins with designing a plastic injection-molded part, followed by the development of rapid tooling and three cooling channels. Concurrently, a mesh model of the geometry is exported, and boundary and initial conditions for the computational analysis are set. Next, the fabrication of the cooling channels and rapid tooling is completed. Numerical simulations are performed, leading to the generation of simulation results. The plastic injection molding process is then conducted with a 900 kN clamping force injection molding machine, and the cooling performance is evaluated; the workflow proceeds by verifying whether the experimental and modeling correlations are satisfied. If discrepancies are found, adjustments are made, and the process is repeated. Once satisfactory results are achieved, a database of high cooling efficiency rapid tooling for the injection molding of LDPE resins is established. Each injection molding experiment was conducted 10 times in this study, and the data represent the average values. This streamlined approach ensures a systematic exploration of cooling efficiency and tool performance in plastic injection molding processes. Ultimately, this study aims to establish a comprehensive database for high-efficiency rapid tooling specifically tailored for the injection molding of LDPE, thereby enhancing the cooling performance and optimizing the overall manufacturing process. Figure 2 depicts a 3D visualization of the four rapid tooling methods. The dimensions of the core and cavity inserts are 120 × 120 × 80 mm, with a Ø15 mm small cylindrical pin used for alignment. Additionally, Ø4 mm holes are incorporated as gate openings. The product thickness of the cup designed in this study is 2 mm. According to the cooling channel design guidelines, a Ø6 mm diameter is selected for the cooling channels to achieve high cooling efficiency and flow distribution. This choice aims to ensure sufficient cooling capacity, reduce cooling time, and enhance both production efficiency and product quality. Figure 3 shows the cooling channel after extracting the support material [28]. Table 1 highlights the key material properties of the rapid tooling utilized in this study for numerical simulation using the Moldex3D simulation software (https://www.moldex3d.com). The heat capacity was measured at 939 J/kg-K, reflecting the material’s ability to store thermal energy. The density of 1.95 g/cm^3^ indicates its compactness. The material exhibits relatively low lateral deformation when subjected to axial stress because aluminum-filled epoxy resin has a Poisson’s ratio of 0.17. The thermal conductivity of 1.1 W/m-K demonstrates the material’s moderate efficiency in heat transfer. Additionally, an elastic modulus of 2.54 GPa indicates the material’s rigidity and resistance to elastic deformation under applied loads. These properties are integral to the performance and functionality of the rapid tooling in this research.

**Figure 4:**
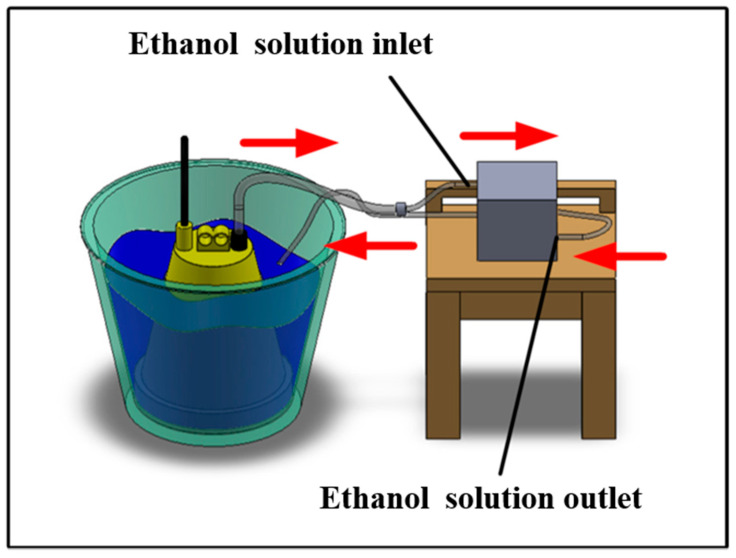
Removal of the cooling channel inside the fast prototype.

**Figure 5:**
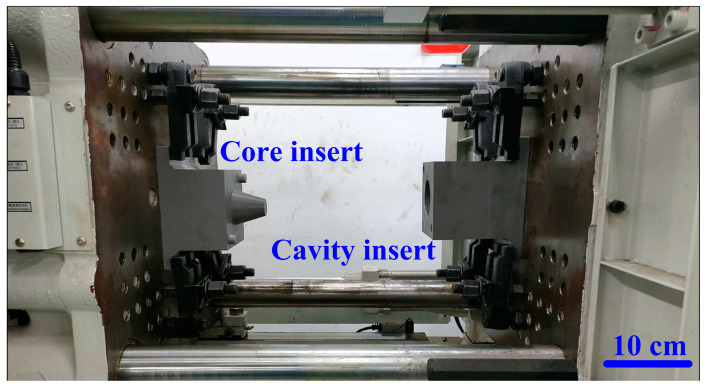
Experimental setup of rapid tooling developed in this work.

**Figure 6:**
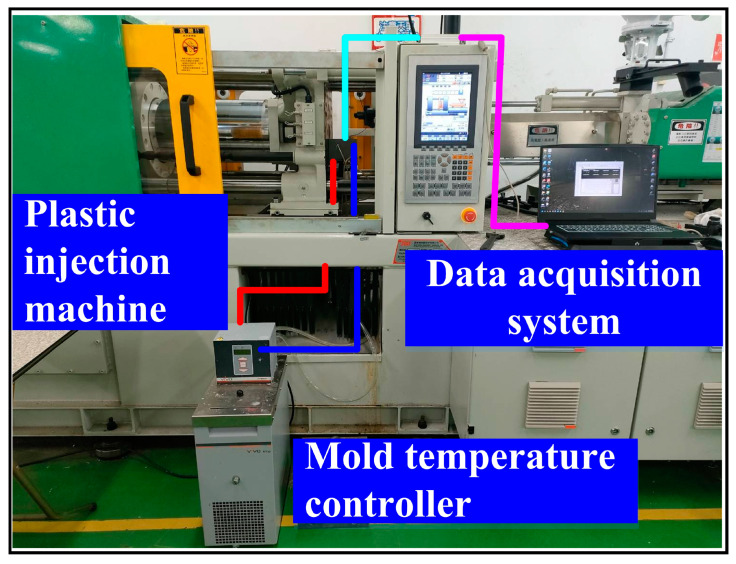
Plastic injection molding designed to evaluate the heat dissipation time of the molded plastic parts using four different rapid tooling methods developed in this work.

**Figure 7:**
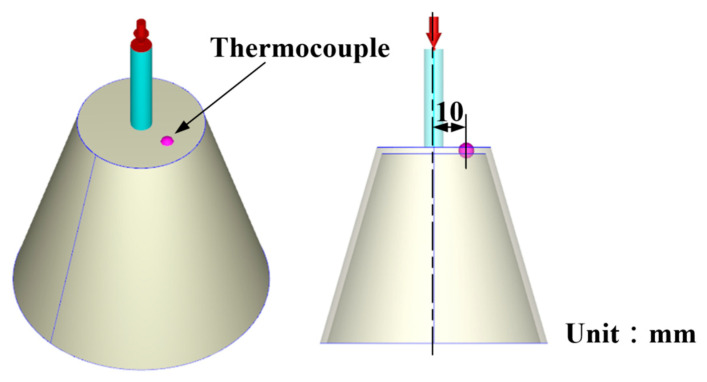
Thermocouple position in the mold.

The additive manufacturing technique enables the fabrication of intricate geometries, such as cooling channels [28]. In this study, a fused deposition modeling apparatus (Teklink solution Inc., New Taipei City, Taiwan) [29] was utilized to fabricate cooling channels using polyvinyl butyral (PVB) filaments [30]. The printing settings for the PVB cooling channels were set with a layer thickness of 0.1 mm, a printing table temperature of 70 °C, a printing speed of 65 mm/s, and a nozzle temperature of 205 °C. A commercial alcohol solution was applied to remove the cooling channels from the rapid tooling. Figure 4 shows the extraction of the cooling channel contained in rapid tooling. Figure 5 shows the experimental setup of the fast prototype developed in this work. Figure 6 depicts the plastic injection molding designed to evaluate the heat dissipation time of the molded plastic parts using four different rapid tooling methods developed in this work. To assess the cooling performance of the fabricated four varying rapid tooling methods, the experimental setup involves K-type thermocouples, a mold temperature controller, and a coolant reservoir integrated with a thermoelectric cooler. The temperature sensors were strategically placed within the mold cavity and linked to a monitoring system for precise monitoring. Ambient conditions were controlled at 27 °C. Figure 7 illustrates the placement of the thermocouple within the mold, positioned 10 mm from the top surface for temperature measurement. The injection pressure, injection time, and packing time are about 10 MPa, 2 s, and 2 s, respectively. Based on a series of tests, the molded parts were ejected once they reached 30 °C. The coolant inlet temperature was maintained at a stable 27 °C throughout the process, and the cooling profiles of the molten LDPE products were continuously monitored and recorded via the temperature sensors. In this study, LDPE [31] was used as a molding material because its non-toxic nature makes it suitable for toys, household products, and medical disposables. Table 2 presents the physical properties of LDPE, obtained via standard testing methods (PAXOTHENE NA207-66, USI Corporation). It has a high melt index, making it especially suitable for injection and extrusion molding. The cooling phase in the injection molding of LDPE parts is critical for successful mass production. Ensuring proper cooling allows for the part to solidify uniformly, preventing issues like warping, shrinkage, and sink marks, which can affect the part’s accuracy and finish. Additionally, the cooling duration impacts cycle times, with shorter times improving production efficiency and output [32]. Effective cooling also helps maintain the integrity of the material, avoiding premature solidification or overcooling that could compromise its properties. Optimizing cooling times leads to lower energy consumption and reduces machine wear, resulting in the cost-effective, high-quality production of LDPE components. To assess the homogeneity of the aluminum (Al) powder within the epoxy resin, the chemical compositions at the top, middle, and bottom sections of the fabricated rapid tooling were analyzed using energy-dispersive X-ray spectroscopy (EDS).

## 3. Results and Discussion

**Figure 8:**
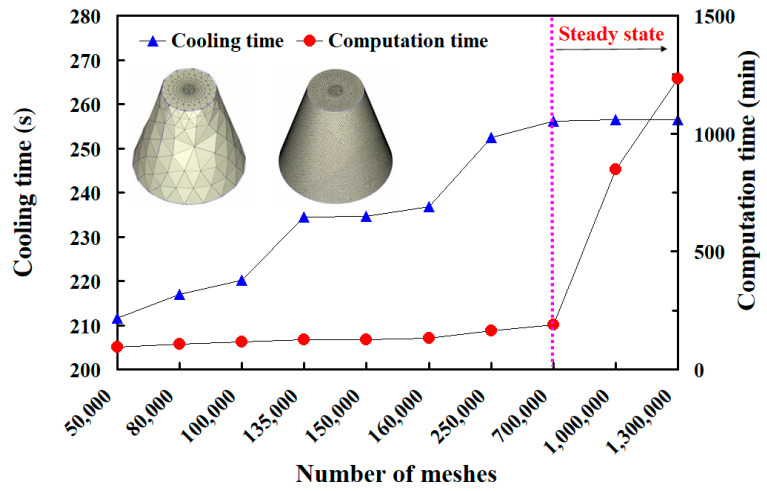
The relationship between the number of meshes and the heat dissipation duration of injection-molded plastic parts.

**Figure 9:**
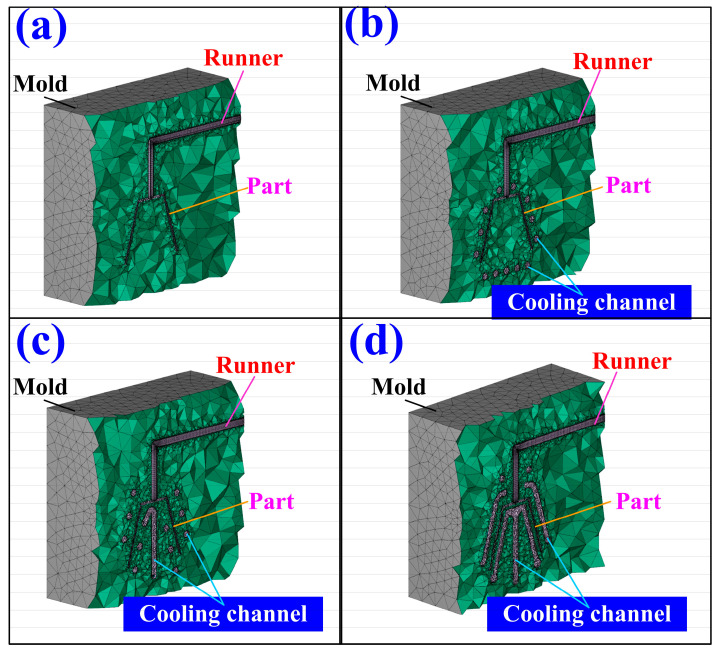
The cross-section view of a rapid tool simulated with BLM for varying cooling channels. (**a**) without cooling channel, (**b**) with conventional cooling channel, (**c**) with conformal cooling channel, and (**d**) with surface-cooled cooling channel.

**Figure 10:**
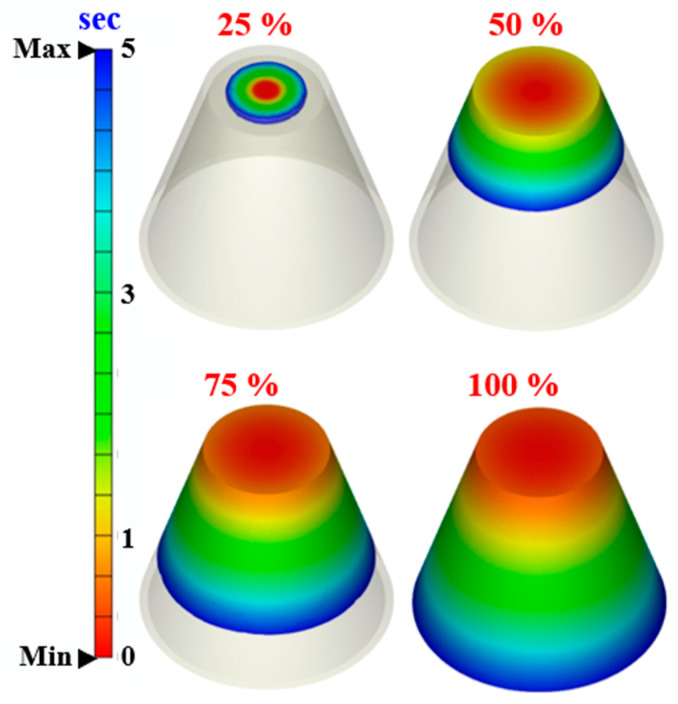
The filling behavior of a molded part.

To determine a suitable mesh size for calculating the heat dissipation time of injection-molded products, this work analyzed ten different mesh sizes. Figure 8 shows the relationships among the number of mesh elements, heat dissipation time, and computational time. The selected mesh sizes were 15 mm, 10 mm, 8 mm, 5 mm, 4 mm, 3 mm, 2 mm, 1 mm, 0.5 mm, and 0.1 mm. These corresponded to approximately 50,000, 80,000, 100,000, 135,000, 150,000, 160,000, 250,000, 700,000, 1,000,000, and 1,300,000 mesh elements, respectively. The calculated cooling times were 211.6 s, 216.9 s, 220.1 s, 234.4 s, 234.7 s, 236.8 s, 252.4 s, 256.2 s, 256.5 s, and 256.4 s. The corresponding computational times were 96 min, 107 min, 118 min, 125 min, 126 min, 133 min, 164 min, 188 min, 847 min, and 1231 min. The results show that smaller mesh sizes increase the number of mesh elements and require longer computational times. When the number of mesh elements reached 700,000, the cooling time stabilized at approximately 256 s. Beyond this point, computational time increased significantly. Higher mesh densities improved simulation accuracy but also required more computational time. To balance accuracy and efficiency, this study used a mesh size of 1 mm to calculate the heat dissipation time of the injection-molded product. This study used solid boundary layer mesh (BLM) for simulations. Figure 9 illustrates the profile of a rapid tool simulated with BLM. The BLM includes a boundary layer with prism elements and an internal layer with tetrahedral elements. Improving the accuracy of viscous heating and pressure results in BLM capturing high shear effects and heat generation reactions. This approach helps reduce warping and deformation. The total mesh count was 387,645 for molds without cooling channels. For rapid tools with conventional cooling channels, the count was 793,653. For rapid tools with conformal cooling channels, it was 693,777. For rapid tools with surface-cooled cooling channels, the total mesh count was 781,670. When performing simulation analysis using Moldex3D, the operator can only set the mesh size, as the software automatically generates the mesh type and cannot be manually modified. The total number of mesh elements is about 167,333. This includes 56,109 tetrahedral elements, 110,328 prism elements, 224 pyramid elements, and 672 hexahedral elements. The gate uses hexahedral elements, which also appear on the contact surface between the product and the gate. Pyramid elements form at the interface between the hexahedral and tetrahedral meshes. The total mesh count for the conventional cooling channel is 438,969. This includes about 307,278 prism elements and 131,691 tetrahedral elements. For the conformal cooling channel, the total mesh count is 320,604, with 224,422 prism elements and 96,182 tetrahedral elements. The surface-cooled cooling channel has a total mesh count of 458,748, consisting of approximately 321,459 prism elements and 137,289 tetrahedral elements. The findings reveal that the conventional cooling channel has a longer path than the conformal cooling channel, resulting in a higher mesh count. The surface-cooled cooling channel uses surface contact cooling, which covers a larger area. As a result, it has the highest mesh count among the three designs. Figure 10 shows the filling behavior of a molded part. The simulation results show that the study model fills within 5 s. After the filling stage, no melt or knit lines appear on the surface of the low-density polyethylene model. This justifies the effectiveness of the system.

**Figure 11:**
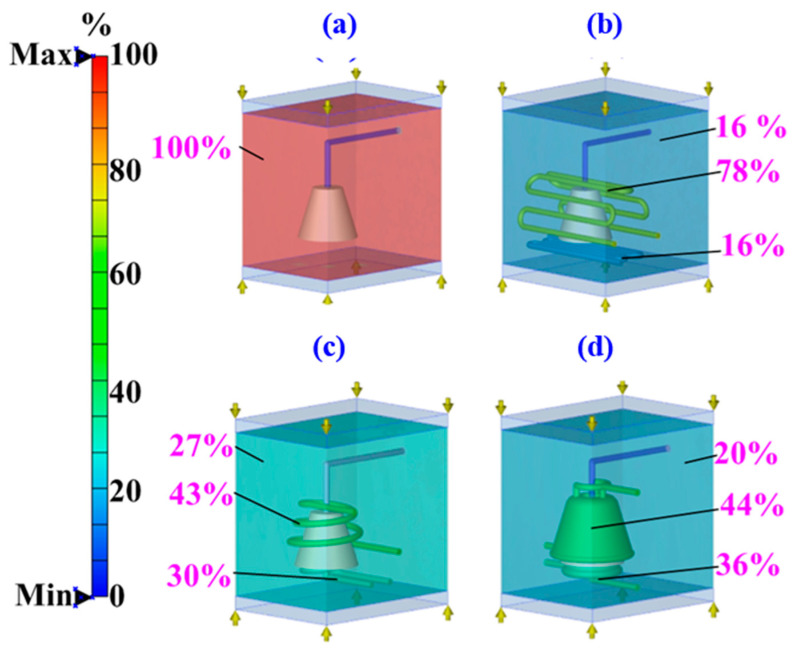
Cooling performance of the core and the cavity for the rapid tooling. (**a**)without cooling channel, (**b**) with conventional cooling channel, (**c**) with conformal cooling channel, and (**d**) with surface-cooled cooling channel.

**Figure 12:**
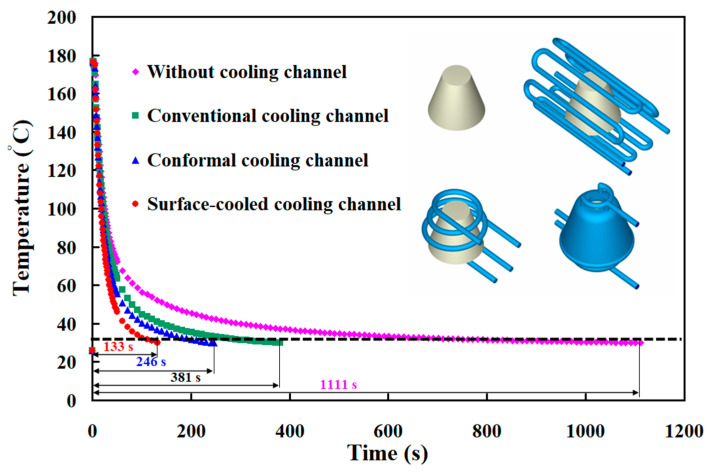
The heat dissipation time of the molded part via the simulation of four different cooling channel setups.

After LDPE plastic injection molding, rapid tooling transfers heat through two main mechanisms: thermal conduction and thermal convection. Thermal convection removes heat into the surrounding environment while cooling channels dissipate heat through conduction. This study analyzed heat transfer in four types of rapid tooling methods to understand their cooling performance. Figure 11 illustrates the cooling performance of the core and the cavity for four rapid tooling methods. The simulation outcomes indicate that rapid tooling without cooling channels relies entirely on the environment for heat dissipation, accounting for 100% of the heat removed. For rapid tooling with conventional cooling channels, heat removal is divided as follows: 16% by the environment, 16% by core cooling channels, and 78% by cavity cooling channels. The difference in heat removed between the core and the cavity is about 62%. For rapid tooling with conformal cooling channels, 27% of heat is removed by the environment, 30% by core cooling channels, and 43% by cavity cooling channels. The difference between the core and the cavity is reduced to 13%. For rapid tooling with SCCC, the proportions are 20%, 36%, and 44%, with the core-to-cavity difference being only 8%. These results show that rapid tooling with SCCC has the smallest difference in heat removal between the core and the cavity. This design leads to more uniform cooling, which improves the quality of the molded parts. A large difference in core and cavity cooling can cause uneven shrinkage and warpage. Therefore, rapid tooling integrated with the SCCC system effectively reduces these defects, thereby enhancing molding precision and overall product quality. Figure 12 shows the heat dissipation duration of the molded part obtained from the four different cooling channel setups. The simulation results reveal that the heat dissipation duration for the rapid mold without cooling channels, from an injection temperature of 180 °C to a demolding temperature of 30 °C, is approximately 1111 s. In comparison, the mold with conventional cooling channels requires about 381 s, the mold with conformal cooling channels takes approximately 246 s, and the mold with surface-cooled cooling channels achieves heat dissipation within only 133 s. The simulation analysis demonstrates that the cooling time varies significantly depending on the cooling system employed. Among the tested designs, the surface-cooled cooling channel exhibits the shortest cooling time, indicating superior cooling performance.

**Figure 13:**
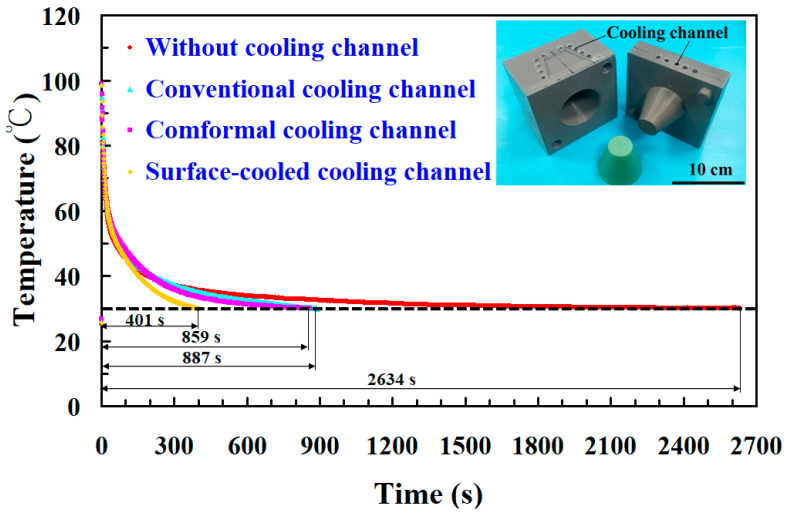
Heat dissipation time of wax patterns produced via low-pressure wax injection using four rapid toolings.

To verify whether the temperature sensors embedded in the four sets of rapid molds can accurately detect the relationship between temperature variations and heat dissipation time of the injection-molded products, this study first conducted experiments using low-pressure wax injection molding. Figure 13 presents the cooling time of wax patterns produced via low-pressure wax injection using four rapid tooling methods. The results indicate that the temperature sensors embedded in the four sets of rapid molds successfully detected the relationship between the temperature variations and the heat dissipation time of the injection-molded products. Based on these data, the heat dissipation time of wax patterns produced through low-pressure wax injection in the four rapid molds can be determined. The findings reveal that the average heat dissipation times of molded products are 2634 s with molds without cooling channels, 887 s with conventional cooling channels, 859 s with conformal cooling channels, and 401 s with surface-cooled cooling channels in low-pressure wax injection molding. These results confirm two critical points: one is that the sensor placement within the rapid mold is precise, and the other is that the sensors reliably capture the temperature-time profile of the molded products, enabling the accurate calculation of average cooling time.

**Figure 14:**
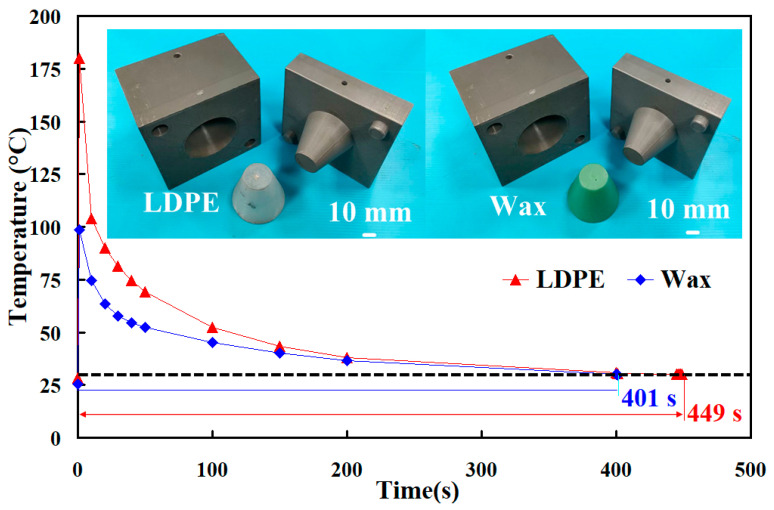
Rapid tooling with the surface-cooled cooling channel, molded LDPE, and wax parts.

**Figure 15:**
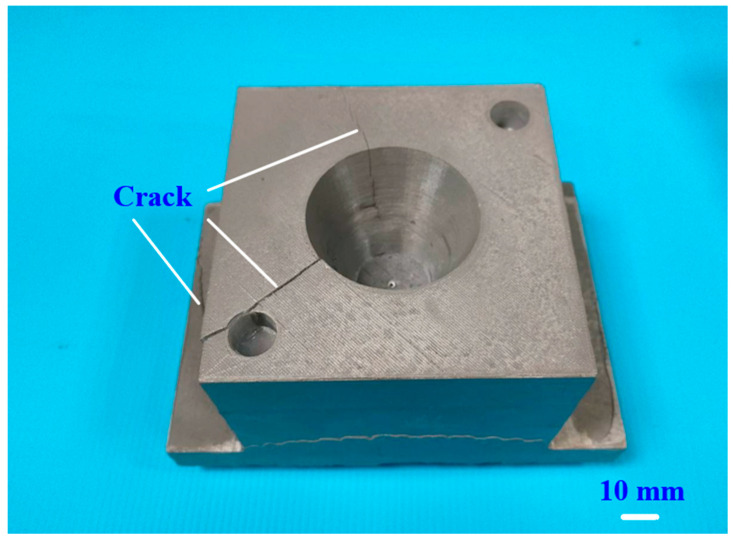
Crack on the rapid tooling after the plastic injection molding of LDPE plastic parts.

To further investigate the feasibility of using rapid tooling in LDPE plastic injection molding, this study employs rapid tooling with a surface-cooled cooling channel to conduct LDPE plastic injection molding processes. Figure 14 shows the rapid tooling with a surface-cooled cooling channel, molded LDPE, and wax parts. Figure 15 shows the crack of the rapid tooling after the plastic injection molding of LDPE plastic parts. The clamping force of a steel mold is approximately 50 MPa. Due to the lower strength of rapid tooling compared to steel molds, the initial experiment in this study set the clamping force at 20 MPa. However, significant cracks were observed in the rapid tooling. To prevent crack formation in aluminum-filled epoxy resin rapid molds in injection molding, several measures can be taken. First, optimizing clamping force reduces overstress on the mold. Second, reinforcing critical mold areas enhances durability [33,34,35]. Subsequent adjustments gradually reduced the clamping force, and when it was lowered to 5 MPa, the rapid tooling showed no cracks and successfully facilitated the injection molding process. To optimize the injection molding of LDPE, several critical parameters must be managed. The melt temperature should be controlled to approximately 160 °C, while the mold temperature is best maintained at approximately 30 °C. Injection pressures are typically approximately 10 MPa, with faster injection speeds required due to LDPE’s excellent flow characteristics. The cooling phase is brief, normally lasting between 20 s. Holding pressures are set at approximately 50% of the injection pressure, with a holding time of approximately 12 s. Low back pressure approximately 1.3 MPa and moderate screw speeds approximately 55 rpm are recommended to ensure proper material plasticization while minimizing the risk of degradation. This combination of parameters helps in achieving precise and efficient molding outcomes.

**Figure 16:**
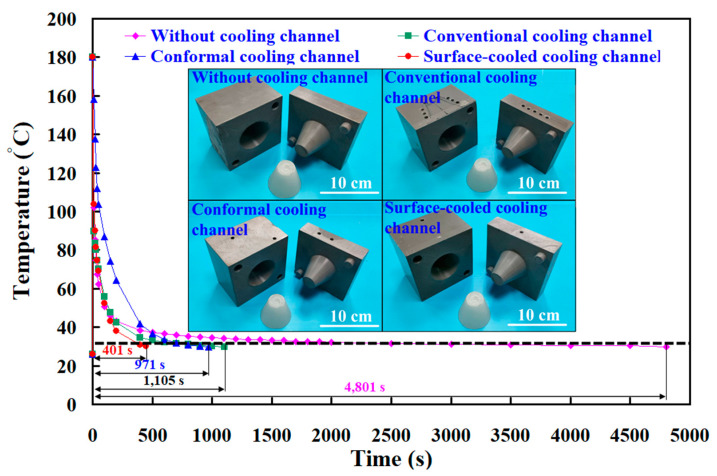
Experiment of the relationship between the heat dissipation time and temperature of the injection molded plastic parts for rapid tooling.

To further understand the cooling time of four rapid tooling molds in LDPE plastic injection molding, this study utilizes four rapid tooling molds to conduct LDPE plastic injection molding experiments. Figure 16 shows the experiment of the relationship between the heat dissipation time and temperature of the injection-molded plastic parts for rapid tooling without a cooling channel, with the conventional cooling channel, with the conformal cooling channel, and with the surface-cooled cooling channel. Experimental results revealed that the cooling times required to cool the molded parts from an injection temperature of 180 °C to a demolding temperature of 30 °C were approximately 4801 s for rapid tooling without cooling channels, 1105 s for rapid tooling with conventional cooling channels, 971 s for rapid tooling with conformal cooling channels, and 401 s for rapid tooling with surface-cooled cooling channels. Two significant findings were obtained: One is that the surface-cooled cooling channel demonstrated superior cooling performance. The other is that its cooling efficiency was approximately 58.7% higher than that of the conformal cooling channel. The use of a surface-cooled cooling channel significantly reduces delivery time for large-scale production. In a 24 h continuous production scenario, the cooling time is reduced to 401 s with the surface-cooled cooling channel, compared to 971 s for the conformal cooling channel. This reduction in cooling time increases the daily production capacity from 84 units with the conformal channel to 204 units with the surface-cooled channel. Consequently, for an order of 1 million units, the production time is shortened from approximately 11,905 days to 4902 days, resulting in a reduction of 7003 days in the delivery period. Furthermore, the surface-cooled cooling channel offers several key advantages for large-scale manufacturing. The shortened cooling time directly reduces energy consumption by minimizing the machine’s operating duration. Additionally, it leads to a reduction in carbon emissions by approximately 2435 tons for an order of 1 million units, compared to the conformal cooling channel. The improved cooling uniformity also minimizes thermal stresses and deformation, ensuring consistent product quality and reducing rejection rates.

**Figure 17:**
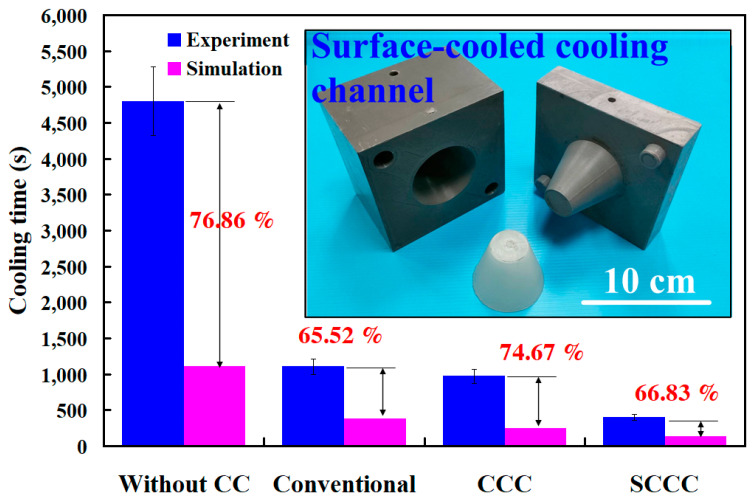
Heat dissipation time of the injection-molded plastic parts for rapid tooling.

**Figure 18:**
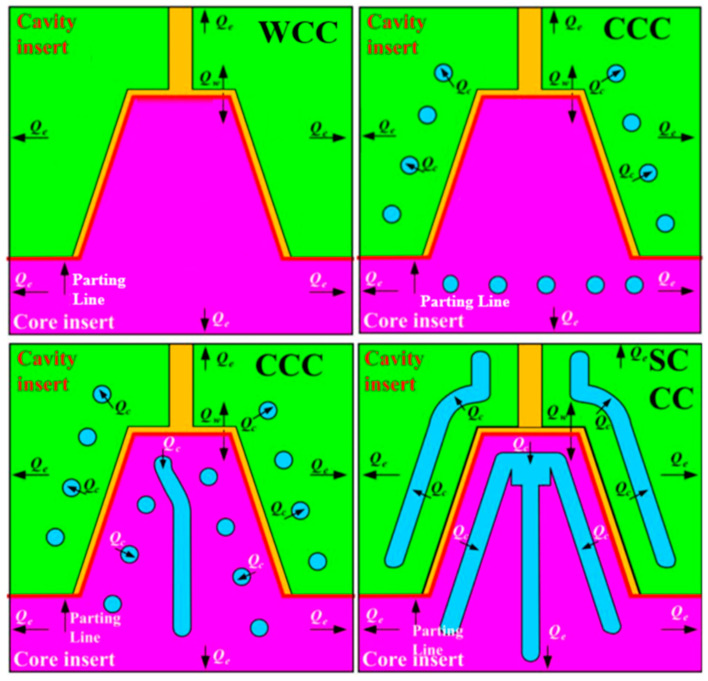
Graphic depiction of the heat fluxes of cooling channels under study, embedded in the rapid tooling in the heat dissipation stage after LDPE injection molding.

Figure 17 shows the heat dissipation time of the injection molded plastic parts for rapid tooling without a cooling channel, with the conventional cooling channel, with the conformal cooling channel, and with the surface-cooled cooling channel. The experimental results revealed discrepancies between the cooling times of injection-molded products obtained through simulations and those measured in practice under various cooling systems. For the rapid tooling without cooling channels, the measured cooling time was approximately 4801 s, while the simulated result was 1111 s, indicating a difference of 3690 s and an error rate of 76.86%. For the rapid tooling with conventional cooling channels, the measured cooling time was approximately 1105 s, compared to a simulated result of 381 s, with a difference of 724 s and an error rate of 65.52%. The rapid tooling with a conformal cooling channel exhibited a measured cooling time of 971 s and a simulated result of 246 s, yielding a difference of 725 s and an error rate of 74.67%. Lastly, the rapid tooling with SCCC showed a measured cooling time of 401 s, with the simulation yielding 133 s, resulting in a difference of 268 s and an error rate of 66.83%. The findings demonstrate that while the simulated and experimental cooling times differ, their trends are consistent. Since the Moldex3D flow analysis software is commonly used for steel molds, this study inputs the parameters of aluminum powder-filled epoxy resin molds and injection molding materials into the software. The simulation results revealed some discrepancies compared to the actual results. However, as both the mold material and injection material were fixed values, the cooling effects of different cooling channels could still be compared effectively. The cooling times ranked from longest to shortest were observed as follows: no cooling channel, conventional cooling channel, conformal cooling channel, and SCCC. Moreover, the error rates between simulations and experimental results ranged from 60% to 80%, indicating that the simulation model provides a degree of predictive reliability. Discrepancies between simulated and experimental cooling times for plastic injection molded parts can arise due to several factors [36]. Variations in mold material properties, molded material properties, mold surface conditions, coolant material properties, and thermal contact resistance significantly impact the cooling time of the molded products. Simulations often assume idealized conditions, potentially overlooking practical variables such as non-uniform mold surfaces or minor variations in cooling channel performance, which lead to deviations from experimental results. Additionally, measurement inaccuracies, including sensor placement and response time, can contribute to observed discrepancies. Together, these factors highlight the need to adjust simulation parameters to match real-world conditions better and enhance predictive accuracy in cooling time assessments. Figure 18 illustrates the graphic depiction of the heat fluxes of cooling channels under study, embedded in the rapid tooling in the cooling stage after LDPE injection molding. Although there is a significant discrepancy between the simulation results and experimental values, the parameters of the mold material and injection material are fixed, allowing for a comparison of the heat flux in different cooling channels. The results show that the heat flux of the SCCC is higher, and it covers a broader range in dissipating heat. SCCC strives to guarantee steady cooling performance for LDPE products after plastic injection molding. By the law of the conservation of energy [37], the equation is obtained as Q_w_ = Q_c_ + Q_e_. Qw, Qc, and Q_e_ represent the heat released from molten LDPE, heat absorbed by the coolant, and heat diffused to the ambient in the cooling stage, respectively. The heat dissipation stage after injection molding involves a complex heat transfer process in the molding procedure [38].

This research presents the advancement of a fast prototype system with high cooling efficiency specifically designed for the injection molding of LDPE. The study optimizes the design of plastic injection molds by incorporating surface-cooled cooling channels, a novel approach that offers significantly improved cooling performance over conventional or conformal cooling systems. The results from this investigation hold considerable practical implications for industrial applications, aligning with the aims of Sustainable Development Goals (SDGs) 7, 9, and 12 [39]. This work fabricated plastic injection molds using aluminum-filled epoxy resin [40] to implement the surface-cooled cooling channel configuration. Furthermore, traditional mold steel [41] can fabricate surface-cooled cooling channels through metal additive manufacturing techniques [42]. Current research efforts are directed toward examining pressure in injection molds, with findings to be reported in forthcoming publications.

## 4. Conclusions

LDPE resin is widely applied in packaging, electrical insulation, industrial liners, and moisture barriers in construction. To enhance cooling performance, this study introduces an innovative SCCC system. Aluminum-filled epoxy resin was used to construct injection molds featuring SCCC, and two mold types were developed. Cooling times were measured using a plastic injection molding machine. A rapid prototype incorporating SCCC was enhanced via Moldex3D simulation to minimize pressure drop. This research ultimately seeks to establish a database for high-efficiency rapid tooling in LDPE injection molding applications. The heat dissipation time of the molded component was analyzed and juxtaposed with the simulated outcomes via the Moldex3D simulation software. This study underscores the promising applications of its findings in industries such as packaging, construction, and electronics, where shorter cooling times can substantially lower production costs in the large-scale manufacturing of LDPE products. The main conclusions derived from the experimental research in this study are as follows:

1. To ensure a balance between computational accuracy and efficiency, a mesh size of 1 mm was employed to evaluate the heat dissipation time of the injection-molded product. The simulation results indicate that the model achieves complete filling within 5 s;
2. EDS analysis showed that aluminum powder was evenly distributed in the epoxy resin matrix, with similar aluminum content across the rapid tooling. This study also explores rapid tooling with surface-cooled cooling channels for LDPE injection molding. The reliable parameters are a melt temperature of 160 °C, a mold temperature of 30 °C, an injection pressure of 10 MPa, and a 20 s cooling time. These conditions help prevent mold cracks and ensure efficient molding;
3. The SCCC aligns with the aims of Sustainable Development Goals, which exhibit enhanced cooling performance, achieving a 58.7% improvement in cooling efficiency compared to the conformal channel. These results in reduced cooling time, increased production capacity, and shortened delivery times. Additionally, it contributes to lower energy consumption, reduced carbon emissions, and a decrease in product defects, offering significant benefits for large-scale manufacturing.

## Figures and Tables

**Table 1:** Properties of the rapid tooling material used in this study.

Parameters	Data
Heat capacity (J/kg-K)	939
Density (g/cm^3^)	1.95
Poisson’s ratio	0.17
Thermal conductivity (W/m-K)	1.1
Elastic modulus (GPa)	2.54

**Table 2:** Physical properties of low-density polyethylene resin (LDPE).

Properties	Test Method	Value
Density (g/cm^3^)	ASTM D1505	0.919
Melt Index (g/10 min)	ASTM D1238	8
Yield Point Tensile Strength (Film) MD/TD	ASTM D882	98/100
Break Point Tensile Strength (Film) MD/TD	ASTM D882	190/135
Ultimate Elongation (Film) MD/TD	ASTM D882	350/480
Impact Strengh (Dart Drop) g/F50	ASTM D1709	90
Haze/Gloss (60°)	ASTM D1003/523	7.0/100
Low Temperature Brittleness (°C/F50)	ASTM D746	<−76
Vicat Softening Point (°C)	ASTM D1525	100
Melt Point (°C)	DSC	109
Hardness (Shore D)	ASTM D2240	530

## Data Availability

The original contributions presented in this study are included in the article. Further inquiries can be directed to the corresponding author.
